# Understanding reasons for unmet health care needs in Korea: what are health policy implications?

**DOI:** 10.1186/s12913-018-3369-2

**Published:** 2018-07-16

**Authors:** Jongnam Hwang

**Affiliations:** Division of Social Welfare and Health Administration, Wonkwang University, Iksan, Korea

**Keywords:** Unmet health care need, Availability, Acceptability, Accessibility, Korea

## Abstract

**Background:**

To ensure equal access to necessary care regardless of an individual’s socioeconomic status, it is crucial to understand the factors that act as barriers. Unmet health care needs can arise for a variety of complex reasons, including personal choice, financial barriers, or lack of services, and each of these reasons requires a different policy approach. Researchers have advocated for a more granular measure of unmet health care need for better policy implication. This study aimed to assess various factors associated with different types of unmet health care needs in Korea.

**Methods:**

The Korean National Health and Nutrition Examination Survey (KNHANES) 2010–2012 was used to analyze responses from 17,610 individuals over age 19. To measure the unmet needs of this population, self-reported experience in the past 1 year was used, and individual’s reasons for unmet need were sorted into three distinct categories – availability, acceptability, accessibility. Four different logistic regression models stratified by gender were used to examine the relationship between socioeconomic factors and unmet needs.

**Results:**

While income was not a significant factor for men, women with lower incomes showed a higher likelihood of experiencing unmet need. In addition, women with lower incomes showed higher odds of having acceptability-related unmet needs during the past 1 year compared to men. Education and income levels were associated with accessibility-related unmet needs for both women and men.

**Conclusion:**

As unmet health care needs are considered to be a critical indicator of a country’s health care system, it is crucial to identify and eliminate any obstacles that prevent access to health care services. Under the current universal health care system in Korea, women, particularly those of lower income and lower educational levels, have limited access to necessary health care services. A gender-specific health care plan is recommended to reduce the higher rate of unmet needs experienced by this group. To reduce accessibility-related unmet needs, increasing available services for younger age groups, reflecting their needs of health services, needs to be considered.

## Background

Ensuring access to necessary care, regardless of the ability to pay, is one of the major determinants of population health [1]. In the past 30 years, Korea has rapidly structured the National Health Insurance (NHI) system, and has achieved universal coverage for all Korean residents [2]. Despite the current universal health care system, out-of-pocket (OOP) spending for health care purposes is higher relative to other Organisation for Economic Cooperative Development (OECD) countries [3]. It has been estimated that annual household OOP spending accounts for approximately 37% of total household health care spending, and the average rate of OOP spending is expected to continuously increase [4]. The increasing financial burden of OOP expenses may cause an uneven distribution of access to necessary services across socioeconomic groups, adversely affecting access and the utilization of health care services by individuals of lower socioeconomic status (SES) [5, 6]. A recent study on unmet health care needs in Korean adults found that adults with the lowest educational attainment reported the greatest difficulty receiving the health care services they needed [7]. Income level was also closely related to health care utilization: those in the lowest income groups were the most likely to report unmet health care needs and those in the highest income groups were the least likely [5]. From a health policy perspective, it is important to understand the factors that act as barriers to receiving health care because failing to access needed services may result in poorer health and ultimately lead to wider gaps in health inequalities [8].

Self-reported unmet needs are a widely accepted indicator of the difficulties in accessing health care services [8–10]. Unmet needs are routinely collected in major national-level surveys, and the Korea National Health and Nutrition Examination Survey (KNHANES) is no exception. Recently, Kim et al. (2015) used KNHANES (wave 4) to explore determinants of unmet need in Korean adults and found that females, young adults, those with lower educational attainment, and those living outside of Seoul were more likely to report having an unmet health care need [7]. Although this study is a good starting point for understanding the driving factors affecting unmet needs, it fails to distinguish between the origins of such needs. Previous research has shown unmet health care needs can arise for a variety of complex reasons, including personal choice, financial barriers, or lack of services, and each reason for experiencing unmet needs requires a different policy approach [8, 11]. Moreover, determinants for each of these reasons for unmet needs are different. For example, higher educational attainment is associated with a greater likelihood of reporting unmet needs because of personal choice; people with a higher education are less likely to report unmet needs due to financial barriers [10]. Accordingly, researchers have advocated for a more granular measure of unmet health care need for better policy implication rather than focusing on accessibility of health care services [8, 9, 11, 12].

Although there is no consensus on the sub-categories of unmet needs, increasing studies have adopted classifying unmet needs into three categories – availability, accessibility, and acceptability in order to identify various determinants of the different reasons for unmet needs [11, 12]. This study adapted this classification in order to fill existing gaps in the current literature. The aim of this study was to identify factors associated with overall unmet needs, as well as three different sub-categories of unmet needs, in Korean women and men.

## Methods

This study used data from the Korean National Health and Nutrition Examination Survey (KNHANES), a national population-based survey that tracks the health of Koreans using three components: a health interview, a nutritional survey, and health examination. A variety of indicators are collected on KNHANES including clinical indicators of health, health behaviours, and socio-demographic characteristics [13]. There are 5 different waves of the KNHANES: 1998, 2001; 2005; 2007–2009; 2010–2012. For the present analyses, this study used data from the 2010–2012 (wave 5) questionnaire components. The total sample of the 2010–2012 survey was 25,534 (male: 11,616 and female: 13,918).

### Dependent variable

The dependent variables were the overall unmet health care needs and the three sub-categories of unmet needs. Overall unmet health care need was measured in response to the question – “*During the past one year, was there ever a time when you felt that you needed health care but didn’t receive it?*”. There were two possible response options: “*Yes*” or “*No*”.

All respondents who indicated they had an unmet need were asked to provide further context, and were requested to answer the question – *“Thinking of your experience of unmet need, why didn’t you get care?”* There were 7 different response options which were sorted into three different categories of unmet need – availability, acceptability and accessibility (Table 1). This classification has been used in previous research of unmet needs using a nationally representative survey with a similarly formatted unmet need-related questionnaire [11, 12]. For those who responded “other” reason and provided an open-ended answer about their reason for unmet need, all responses for “other” reason with an open-ended answer were reviewed and re-classified into one of the three main categories (availability, acceptability and accessibility).

**Table 1:** Classification of unmet health care need and percentage of self-reported unmet needs from the Korea National Health and Nutrition Examination Survey (KNHANES) 2010–2012

Category of unmet need^a^	Stated reasons for unmet need^a^	n (%)
Availability	Waiting time too long	1139 (6.47)
	Not available when required	
	Not available in area	
Acceptability	Felt it would be inadequate	1242 (7.05)
	Didn’t get around to it	
	Decided not to seek care	
	Too busy	
	Didn’t know where to go	
	Dislike doctors/afraid	
	Personal/Family responsibilities	
Accessibility	Cost	811 (4.61)
	Transportation	

^a^Classification of unmet needs was adopted from Hou & Chen [12]

### Predictor variables

This study used the Andersen’s Health Behaviour Model (HBM) and previous studies to guide the selection of potential predictor variables [14]. The HBM is a framework that is designed to assist in the understanding of the determinants of health services use, and has been widely used for health services studies that examine access to and the utilization of health care [15]. According to Andersen’s HBM, the determinants of health care utilization can be classified into three domains: (1) predisposing characteristics, (2) enabling resources, and (3) need-based factors [14]. Predisposing factors are generally demographic factors associated with the use of health care services such as sex, age, educational attainment, and other socio-demographic factors. Enabling resources refer to factors available to individuals that facilitate their use of health care services, such as insurance coverage, the availability of physicians, and income. Need-based factors are indicators of actual health status that are often linked to the use of health care services**.**

In the present study, age (i.e., 19–34, 35–49, 50–64, 65+), marital status (i.e., married or living with partner vs. single or living alone) and educational attainment (i.e., completion of elementary school, middle school, high school, and post-secondary) were included as predisposing factors. Self-reported income, insurance status – NHI or Medicaid recipient, and place of residence were included as enabling factors. Medicaid is the health care program for the poor, funded and operated by both the central and local governments in Korea. For place of residence, unmet need in four different regions were compared: Seoul, Incheon/Gyeonggi, Chungcheong (including Daejeon)/Gangwon, Jeolla (Gwangjoo included)/Jeju and Gyeongsang (including Busan, Daegu, and Ulsan). Seoul is the country’s capital city, where approximately 10 million people dwell, accounting for 20% of the national population. Because nearly half of the country’s population is concentrated in the Metro Seoul regions, including Seoul and Incheon/Gyeonggi, uneven distribution of health care resources has been identified as a major challenge in Korea [6, 16, 17].

For the need-based factor, a self-rated health assessment was used as an indicator. Individuals were asked to rate their general health and their original responses were “Excellent, very good, good, fair, poor and very poor”. The original responses were re-classified into 3 categories – Good (including excellent, very good, good), fair, and poor (including poor and very poor).

Four separate analyses were conducted to examine the factors associated with overall unmet health care needs, as well as accessibility-, availability-, and acceptability- related unmet needs, respectively. All descriptive and multiple logistic regressions were conducted using STATA v.12. To understand the different impacts of unmet health care needs on women and men, all analyses were stratified by gender. Results are presented as odds ratio (OR) with 95% CIs and *p*-value. The survey weights provided by the KNHANES were applied for all analyses, to account for the complex survey design and sample selection, as well as to represent the actual Korean population [13].

## Results

There were 17,610 adults included in this study, which yielded an estimated population of 37,814,657 after applying survey weights. Of the 18,735,934 women included, 4,204,621 (22.4%) reported experiencing an unmet health care need within the past 1 year. Of the 19,078,723 men included 2,622,189 (14.5%) reported experiencing an unmet health care need in the past year. Table 2 presents general characteristics of the estimated population by overall unmet health care needs. Approximately 26% of both the youngest (19–34) and oldest (65 +) age groups of women experienced unmet health care needs, which were the highest rate in women. In addition, the younger (19–34 and 35–49) groups of men reported the highest number of experiencing unmet needs. Compared to individuals who were married or living with a partner, individuals who were single or living alone reported a slightly higher rate of unmet needs (Women: 20.1% vs. 27.4%; men: 14.4% vs. 14.8%, respectively). The lowest educational attainment groups in both women and men had higher rates of unmet needs (27.4 and 16.1%, respectively) compared to individuals with higher educational attainment. Women with the lowest income showed the highest rate of unmet health care needs (30.7%) while the rate of unmet needs in men did not significantly vary across income quartile groups. In regard to occupation, the highest rate of unmet need in women was observed in the farming and fishing industry (28.1%). In men, those with service and sales jobs reported the highest rate of not receiving necessary health care services (16.7%). Both women and men living in the Busan, Daegu, Ulsan & Gyeongsang provinces reported more experiences of unmet needs compared to other regions (28.6 and 21.4%, respectively). Medicaid recipients and individuals with poor self-rated health showed the highest rate of experiencing unmet needs.

**Tables 2:** Descriptive characteristics of the sample by overall unmet health care needs, KNHANES 2010–2012

Variables		Overall unmet need					
		Female			Male		
		Yes - Estimated n (%)	No- Estimated n (%)	*p*-value^*^	Yes - Estimated n (%)	No- Estimated n (%)	*p*-value^*^
Unmet need	Experience within the past 1 year	4,204,621 (22.4)	14,531,313 (77.6)		2,622,189(14.5)	15,456,535 (85.5)	
Age	19–34	1,369,050 (26.0)	3,898,168 (74.0)	< 0.01	830,225 (15.0)	4,719,006 (85.5)	0.16
	35–49	1,062,884 (17.9)	4,860,195 (82.1)		924,922 (15.3)	5,124,277 (84.7)	
	50–64	962,174 (21.2)	3,569,141 (78.8)		619,790 (14.2)	3,735,121 (85.8)	
	65 +	810,513 (26.9)	2,203,809 (73.1)		247,251 (11.6)	1,878,132 (88.4)	
Marital status	Married or living with partner	2,522,832 (20.1)	10,017,217 (79.9)	< 0.01	1,801,493 (14.4)	10,729,838 (85.6)	0.74
	Single or living alone	1,681,790 (27.1)	4,514,095 (72.9)		820,696 (14.8)	4,726,697 (85.2)	
Education	Completion of elementary school	1,296,794 (27.4)	3,434,929 (72.6)	< 0.01	354,495 (16.1)	1,843,690 (83.9)	0.28
	Completion of middle school	387,824 (20.5)	1,502,759 (79.5)		272,357 (14.8)	1,563,496 (85.2)	
	Completion of high school	1,338,885 (19.8)	5,438,113 (80.2)		1,128,855 (15.0)	6,380,613 (85.0)	
	Completion of post-secondary	1,181,118 (22.1)	4,155,511 (77.9)		866,481 (13.3)	5,668,736 (86.7)	
Income	Q1(Lowest)	1,044,038 (30.7)	2,355,908 (69.3)	< 0.01	344,707 (13.7)	2,173,829 (86.3)	0.71
	Q2	1,177,407 (22.7)	4,014,225 (77.3)		733,115 (15.2)	4,087,629 (84.8)	
	Q3	1,112,956 (21.4)	4,084,109 (78.6)		819,758 (14.9)	4,695,497 (85.1)	
	Q4(Highest)	870,221 (17.6)	4,077,071 (82.4)		724,608 (13.9)	4,499,580 (86.1)	
Occupation	White collar and office	908,711 (25.9)	2,598,568 (74.1)	< 0.01	697,074 (13.5)	4,460,213 (86.5)	0.01
	Services and sales	641,635 (22.6)	2,195,116 (77.4)		396,073 (16.7)	1,981,006 (83.3)	
	Farming & fishing	263,807 (28.1)	674,658 (71.9)		219,529 (14.9)	1,249,880 (85.1)	
	Blue collar	574,624 (24.7)	1,753,012 (75.3)		848,462 (16.5)	4,296,984 (83.5)	
	Out of labour market (Homemakers/students, etc.)	1,815,844 (19.9)	7,309,959 (80.1)		461,051 (11.7)	3,468,453 (88.3)	
Region	Seoul	743,067 (18.6)	3,253,174 (81.4)	< 0.01	396,202 (10.6)	3,339,972 (89.4)	< 0.01
	Incheon & Gyeonggi	1,054,958 (19.9)	4,252,994 (80.1)		661,425 (12.7)	4,544,040 (87.3)	
	Daejeon & Chungcheong/ Gangwon	544,751 (22.2)	1,912,552 (77.8)		302,217 (12.2)	2,180,618 (87.8)	
	Gwangjoo & Jeolla/ Jeju	470,726 (22.3)	1,635,504 (77.7)		275,190 (13.5)	1,760,897 (86.5)	
	Busan, Daegu, Ulsan & Gyeongsang	1,391,120 (28.6)	3,477,089 (71.4)		987,155 (21.4)	3,631,009 (78.6)	
Health insurance	National Health Insurance (NHI)	3,974,949 (22.0)	14,088,971 (78.0)	< 0.01	2,543,768 (14.5)	15,041,721 (85.5)	0.14
	Medicaid	203,661 (35.2)	375,146 (64.8)		711,59 (19.9)	286,956 (80.1)	
	No & don’t know	26,010 (27.9)	67,195 (72.1)		7262 (0.4)	127,858 (94.6)	
Self-rated health	Very good/good	875,945 (15.3)	4,857,410 (84.7)	< 0.01	747,844 (10.7)	6,214,591 (89.3)	< 0.01
	Fair	1,926,262 (21.3)	7,106,279 (78.7)		7,404,775 (14.1)	1,219,802 (85.9)	
	Poor/Very poor	1,402,414 (35.3)	2,567,624 (64.7)		654,543 (26.3)	1,837,170 (73.7)	

^*^The *p*-values in the univariate analyses were chi-squares (for two categorical variables) and ANOVA (for multiple categorical variables), indicating the relationship between the dependent variable and independent variables

Table 3 and Table 4 present the results from the gender-stratified logistic regression models, examining factors associated with overall unmet health care need and the three sub-categories of unmet need in Korean women and men. With respect to overall unmet health care need, the youngest age group (19–34) of women were more likely to have unmet needs compared to the older age groups (OR: 1.55 95%CI: 1.19–2.01). In men, the younger age groups, in particular age 19–34 and 35–49, were also more likely to experience unmet needs compared to the older age groups (OR: 1.72 95%CI: 1.22–2.44; OR: 1.54 95%CI: 1.13–2.11, respectively). While income was not a significant determinant in men, women with lower household income (Q1, Q2 and Q3) showed higher odds of experiencing unmet needs (OR: 1.78 95%CI: 1.42–2.24; OR: 1.39 95%CI: 1.16–1.67; OR: 1.27 95%CI: 1.07–1.51). A higher likelihood of reporting unmet needs was also observed across the younger groups (19–34, 35–49, and 50–64) in both women and men in the availability-related unmet need models. (Women = OR: 4.73 95%CI: 3.00–7.46; OR: 2.35 95%CI: 1.55–3.56; OR: 2.43 95%CI: 1;65–3.59; Men = OR: 3.70 95%CI: 2.02–6.77; OR: 2.57 95%CI: 1.52–4.36; OR: 2.03 95%CI: 1.20–3.43, respectively). With respect to acceptability-related unmet needs, women with lower incomes (Q1 and Q2) showed higher odds of having acceptability-related unmet needs during the past 1 year. In contrast, income level was not associated with a higher likelihood of experiencing acceptability-related unmet needs in men. Both educational and income levels were associated with higher odds of having accessibility-related unmet needs in women and men. In women, lower educational attainment, when compared to the highest educational attainment, had higher odds of experiencing accessibility-related unmet needs (OR: 2.61 95%CI: 1.54–4.43; OR: 1.95 95%CI: 1.06–3.58; OR: 1.58 95%CI: 1.01–2.46). In men, completion of elementary school and high school were associated with a greater likelihood of having accessibility-related unmet needs (OR: 3.41 95%CI: 1.39–8.36; OR: 2.27 95%CI: 1.13–4.58, respectively). Across all household income groups (Q1, Q2, and Q3), a higher likelihood of experiencing an accessibility-related unmet need was consistently observed in both women and men, although the odds ratio was lower when it came to higher educational levels. (Women = OR: 4.80 95%CI: 2.95–7.80; OR: 2.66 95%CI: 1.71–4.14; OR: 1.63 95%CI: 1.02–2.61; Men = OR: 3.30 95%CI: 1.28–8.49; OR: 2.96 95%CI: 1.22–7.21; OR: 2.55 95%CI: 1.04–6.28).

**Tables 3:** Odds and 95% CIs from logistic regression models examining socioeconomic factors and overall unmet need and reasons for unmet health care needs (availability, acceptability, and accessibility) among women, KNHANES 2010–2012

Variables	Overall unmet need (weighted n = 18,735,934)			Availability unmet need (weighted n = 16,013,627)			Acceptability unmet need (weighted n = 16,145,912)			Accessibility unmet need (weighted *n* = 15,639,020)		
	OR	95%CI	*p*-value	OR	95%CI	*p*-value	OR	95%CI	*p*-value	OR	95%CI	*p*-value
Age (Ref: 65+)												
19–34	1.55	1.19–2.01	< 0.01^*^	4.73	3.00 - 7.46	< 0.01^*^	1.19	0.81 - 1.75	0.38	1.19	0.73–1.93	0.49
35–49	0.99	0.76–1.29	0.93	2.35	1.55–3.56	< 0.01^*^	0.91	0.63 - 1.31	0.62	0.89	0.54–1.45	0.64
50–64	1.00	0.82–1.22	0.99	2.43	1.65–3.59	< 0.01^*^	0.82	0.63 - 1.08	0.38	1.01	0.74–1.40	0.94
Marital status (Ref: Married or living with partner)												
Single or living alone	1.12	0.97–1.29	0.12	1.25	1.02–1.54	0.03^*^	0.86	0.68 - 1.07	0.18	1.34	1.03–1.74	0.03^*^
Education (Ref: Post-secondary)												
Completion of elementary school	1.19	0.91–1.55	0.20	1.09	0.71–1.67	0.70	0.83	0.57–1.22	0.35	2.61	1.54–4.43	< 0.01^*^
Completion of middle school	0.98	0.72–1.32	0.88	0.82	0.49–1.39	0.46	0.81	0.55–1.18	0.27	1.95	1.06–3.58	0.03^*^
Completion of high school	0.89	0.74–1.07	0.21	0.91	0.69–1.20	0.50	0.74	0.57–0.96	0.02^*^	1.58	1.01-2.46	0.04^*^
Annual household income (Ref: Q4; Highest)												
Q1	1.78	1.42–2.24	< 0.01^*^	1.12	0.76 - 1.64	0.58	1.36	1.01–1.85	0.04^*^	4.80	2.95-7.80	< 0.01^*^
Q2	1.39	1.16 - 1.67	< 0.01^*^	1.02	0.76 - 1.35	0.91	1.44	1.14–1.81	< 0.01^*^	2.66	1.71-4.14	< 0.01^*^
Q3	1.27	1.07 - 1.51	< 0.01^*^	1.18	0.92 - 1.50	0.18	1.27	0.99–1.63	0.06	1.63	1.02–2.61	0.04^*^
Occupation (Ref: White collar/Office)												
Services & Sales	0.85	0.67–1.06	0.15	0.76	0.57–1.02	0.07	1.00	0.57–1.02	0.07	1.17	0.70–1.94	0.55
Farming & Fishing	0.87	0.64–1.19	0.38	0.92	0.59–1.42	0.71	1.52	0.59–1.42	0.71	0.71	0.36–1.38	0.31
Blue collar	0.85	0.67–1.08	0.18	0.88	0.63–1.24	0.47	1.18	0.63–1.24	0.47	0.87	0.50–1.49	0.60
Out of labour market (Homemakers/students, etc.)	0.59	0.49–0.71	< 0.01^*^	0.24	0.18 - 0.32	< 0.01^*^	1.20	0.18 - 0.32	< 0.01^*^	0.84	0.54-1.32	0.46
Region (Ref: Seoul)												
Incheon & Gyeonggi	1.09	0.91–1.30	0.35	0.87	0.66–1.15	0.34	1.33	1.03–1.70	0.03^*^	1.09	0.78-1.52	0.63
Daejeon & Chungcheong/ Gangwon	1.14	0.92–1.41	0.24	1.17	0.81–1.70	0.39	1.20	0.87–1.65	0.27	1.06	0.74–1.51	0.75
Gwangjoo & Jeolla/ Jeju	1.11	0.87–1.43	0.39	1.21	0.89–1.65	0.22	0.91	0.61–1.38	0.66	1.11	0.72–1.71	0.62
Busan, Daegu, Ulsan & Gyeongsang	1.66	1.39–1.99	< 0.01^*^	1.30	0.97 - 1.74	0.08	2.38	1.85–3.07	< 0.01^*^	1.23	0.90-1.68	0.19
National Health Insurance (Ref: Yes)												
Medicaid	1.30	0.94–1.80	0.12	1.35	0.67–2.73	0.84	1.16	0.67–2.01	0.60	1.30	0.87–1.94	0.20
No & don’t know	0.97	0.43–2.16	0.93	0.96	0.23–3.97	0.95	0.96	0.32–2.88	0.95	0.97	0.28–3.44	0.97
Self-rated health (Ref: Good)												
Fair	1.45	1.26–1.75	< 0.01^*^	1.42	1.10 - 1.85	< 0.01^*^	1.52	1.20-1.91	< 0.01^*^	1.50	1.06-2.13	0.02^*^
Poor	2.87	2.37 - 3.47	< 0.01^*^	2.72	1.96 - 3.77	< 0.01^*^	2.11	1.63-2.73	< 0.01^*^	4.49	3.17-6.36	< 0.01^*^

^*^p < 0.05

**Table 4:** Odds and 95% CIs from logistic regression models examining socioeconomic factors and overall unmet need and reasons for unmet health care needs (availability, acceptability, and accessibility) among men, KNHANES 2010–2012

Variables	Overall unmet need (weighted *n* = 18,078,724)			Availability unmet need (weighted *n* = 16,784,081)			Acceptability unmet need (weighted n = 16,207,830)			Accessibility unmet need (weighted *n* = 15,872,025)		
	OR	95%CI	*p*-value	OR	95%CI	*p*-value	OR	95%CI	*p*-value	OR	95%CI	*p*-value
Age (Ref: 65+)												
19–34	1.72	1.22–2.44	< 0.01^*^	3.70	2.02 - 6.77	< 0.01^*^	1.21	0.66-2.20	0.54	1.34	0.59–3.00	0.48
35–49	1.54	1.13–2.11	< 0.01^*^	2.57	1.52 - 4.36	< 0.01^*^	1.04	0.99-2.85	0.05	1.03	0.54–1.96	0.93
50–64	1.18	0.91–1.54	0.20	2.03	1.20–3.43	< 0.01^*^	1.26	0.83-1.89	0.27	0.88	0.52–1.51	0.66
Marital status (Ref: Married or living with partner)												
Single or living alone	1.05	0.81–1.35	0.72	0.95	0.68–1.34	0.78	1.38	0.01–2.09	0.13	0.79	0.42–1.46	0.45
Education (Ref: Post-secondary)												
Completion of elementary school	1.41	1.00–1.99	0.05	0.87	0.51–1.46	0.59	1.39	0.79–2.44	0.25	3.41	1.39–8.36	< 0.01^*^
Completion of middle school	1.20	0.83–1.70	0.34	1.07	0.67–1.72	0.77	1.04	0.59–1.82	0.90	2.32	0.93–5.75	0.07
Completion of high school	1.15	0.92–1.45	0.21	1.16	0.88–1.54	0.30	0.94	0.66–1.35	0.75	2.27	1.13–4.58	0.02^*^
Annual household income (Ref: Q4; Highest)												
Q1	0.88	0.64–1.22	0.45	0.61	0.33–1.12	0.11	0.85	0.53–1.37	0.50	3.30	1.28–8.49	0.01^*^
Q2	1.00	0.78 - 1.27	0.98	0.74	0.54–1.01	0.05	1.21	0.80–1.82	0.36	2.96	1.22–7.21	0.02^*^
Q3	1.03	0.82 - 1.29	0.82	0.88	0.67–1.15	0.35	1.08	0.71–1.65	0.72	2.55	1.04–6.28	0.04^*^
Occupation (Ref: White collar/Office)												
Services & Sales	1.18	0.89–1.57	0.25	1.06	0.74–1.52	0.76	1.82	1.16–2.85	0.01^*^	0.55	0.19-1.59	0.27
Farming & Fishing	0.98	0.69–1.39	0.89	0.88	0.56–1.38	0.57	1.73	1.00–3.00	0.05	0.71	0.23–2.24	0.56
Blue collar	1.12	0.88–1.42	0.37	1.14	0.84–1.55	0.40	1.15	0.73–1.80	0.54	1.24	0.57–2.67	0.58
Out of labour market (Homemakers/students, etc.)	0.70	0.52–0.94	0.02	0.27	0.15–0.48	< 0.01^*^	1.28	0.80-2.08	0.31	1.25	0.56–2.80	0.58
Region (Ref: Seoul)												
Incheon & Gyeonggi	1.13	0.86–1.47	0.38	1.23	0.88–1.71	0.22	1.24	0.76–2.05	0.39	0.75	0.45–1.25	0.27
Daejeon & Chungcheong/ Gangwon	1.06	0.74–1.50	0.76	0.90	0.54–1.50	0.68	1.33	0.71–2.49	0.38	1.01	0.49–2.08	0.99
Gwangjoo & Jeolla/ Jeju	1.18	0.76–1.85	0.47	1.49	0.83–2.68	0.18	1.26	0.70–2.28	0.45	0.52	0.23–1.16	0.11
Busan, Daegu, Ulsan & Gyeongsang	2.21	1.73–2.82	< 0.01^*^	2.25	1.62 - 3.12	< 0.01^*^	3.60	2.30-5.63	< 0.01^*^	0.72	0.42-.1.24	0.23
National Health Insurance (Ref: Yes)												
Medicaid	1.30	0.71–2.37	0.39	0.36	0.08–1.69	0.19	1.45	0.66–3.20	0.36	1.69	0.67–4.27	0.26
No & don’t know	0.37	0.08–1.75	0.21	0.68	0.09–5.12	0.71	1.00	–	–	0.43	0.06–3.29	0.41
Self-rated health (Ref: Good)												
Fair	1.34	1.07–1.68	0.01^*^	1.67	1.26 - 2.22	< 0.01^*^	1.05	0.75-1.48	0.77	1.03	0.59–1.78	0.93
Poor	3.36	2.58–4.37	< 0.01^*^	3.76	2.60 - 5.43	< 0.01^*^	2.22	1.50-3.29	< 0.01^*^	4.37	2.48-7.71	< 0.01^*^

^*^p < 0.05

## Discussion

Despite the promise of universal health care in Korea, this study found that a substantial proportion of Koreans had unmet health care needs; this was particularly apparent among people of a lower socioeconomic status (SES). The results also show that individual factors affecting unmet needs vary by gender and by the reasons for the unmet needs. To the author’s knowledge, this study is the first attempt to classify the reasons for unmet health care needs in Korea based on the pervasive classification (i.e., availability-, acceptability, and accessibility-related unmet need) and provides a more complete picture of the problem.

Economic status, commonly represented by income levels, has been thought to be a major risk factor for reporting unmet health care needs, and increasing number of studies suggest policy actions for minimizing economic barriers affecting access to necessary care [18–22]. In this study, the results indicate that income is associated with unmet health care needs. In particular, lower income is associated with higher overall unmet needs in women but not men, and lower income is associated with higher accessibility-related unmet needs in both men and women. These findings are in direct opposition to *Kim* et al’s recent study on unmet needs [7]. In the previous study, there was insufficient evidence to suggest income impacted unmet health care needs. Part of these differences may be attributed to *Kim* et al’s decision to pool men and women together as well as their failure to stratify the analyses by the type of unmet health care need.

It is understandable that women and men may experience unmet needs differently. Women, in general, earn lower incomes and have lower-paying positions at work than men, and consequently have more difficulties arranging and attending appointments, accessing necessary services, and communicating their health issues to health professionals [23]. For women who stay at home, familial responsibilities may interfere, preventing them from accessing and utilizing needed health care services [23]. An increased probability of experiencing unmet needs by women in lower-income households suggests more attention be given to developing a gender-sensitive health policy to meet the needs of women with lower incomes [24].

Reasons for unmet health care needs are complex and multi-dimensional, but most existing studies consider an individual’s experience of unmet health care needs as a barrier to accessing health care services [8, 9]. Aggregated self-reported assessments of unmet needs can be a good starting point, but are likely to be considered inadequate for the purpose of developing an effective policy that will truly minimize unmet needs [9]. From this perspective, the results from the three main categories for unmet health care needs point to different policy implications. In relation to accessibility, which encompasses cost and transit related barriers, household income and educational levels were key determinants of unmet need, wherein men and women of the lowest income and educational attainment reported the highest odds of unmet needs. This finding implies that individuals of lower socioeconomic status have more barriers to accessing necessary care compared to their counterparts. Despite successful implementation of Korea’s universal health care system, economic barriers to receiving health services remain [25]. In fact, existing studies of Korean populations suggest that income and educational levels are associated with having a higher probability of experiencing unmet needs along with gender, age, health status, and occupation [5, 26, 27]. This observed relationship between lower SES and experiencing unmet needs is not unique to Korea. Other countries with universal health care systems also report income-related differences in unmet health care needs [12, 28].

To reduce accessibility-related barriers, expanding the current National Health Insurance (NHI) coverage could be a viable option. Previous studies demonstrated that the continuous reforms over the past decades that expanded benefit coverage of the NHI has led to an overall improvement in access to care and service utilization, although higher OOP spending still exists as a possible financial barrier [29–33]. For example, a recent study using the NHI claims data found that, after expanding the NHI benefit coverage of cancer-related services, health services utilization for outpatient and inpatient care increased more in the low-income groups than in the high-income groups [29]. This suggests the relationship between the NHI benefit coverage and access to care provides policy implications that accessibility-related unmet health care needs in lower SES groups could be improved by diminishing financial barriers.

The older age groups were less likely to experience availability-related unmet need compared to the youngest age group in both women and men. Although younger adults are expected to have less health problems compared to older adults, the younger age groups consistently reported more availability-related unmet need across different countries [34, 35]. This may imply that younger people have a higher expectation about health care services, so they are more likely express their dissatisfaction when services are not available [36–39]. In addition, it is also plausible that younger people tend to be more pro-active about seeking health care services when required, so they may encounter more unavailability of the services they need [40]. Also, marital status was an important factor associated with availability-related unmet health care needs in women. Women who live alone are more likely to express difficulties in finding available health care resources compared to men. In current literature, social support and social capital are closely related to health care utilization and unmet health care needs [23, 41]. One possible explanations is that women living alone have limited social supports, resulting in limited information on where and how to access the services they need [35]. It is also plausible that a larger number of single women contact the health care system in order to seek health care services, which may increase more chances of encountering service unavailability.

Previous studies have suggested that acceptability-related unmet health care needs generally stem from personal preference or circumstance [11, 12]. Due to this quandary, the results are difficult to interpret and policy implications to address this category of unmet need are not clear [11]. However, it is worthwhile to note that acceptability-related unmet needs may be associated with future experiences of, and responses to health care, one of fundamental dimensions of the system effectiveness [42]. Therefore, further research to understand acceptability-related problems needs to be prioritized. In addition, understanding how regional differences affect experiences of unmet need is important considering there is an increasing concern regarding regional inequalities in health and health care. For instance, the results from this study indicate that both women and men living in Busan, Dageu, Ulsan, and Gyeonsang province have higher odds of reporting an overall unmet health care need. Although multiple factors determine an individual’s experience of unmet health care needs, the higher concentration of health care facilities in the Metro-Seoul regions may be driving these regional differences [16, 43]. Regional inequalities in health outcomes and health care utilization have become an increasing concern in Korea and further scrutiny is considered to fully understand how regional differences affect unmet needs and future policy development [44].

### Limitations

Some limitations of this study have been identified. The KNHANES is a cross-sectional survey, and the outcome–unmet needs – corresponds to the unmet needs reported in the year prior to the survey. On the other hand, the predictor variables may correspond to the date the data was collected; it is not clear if these predictor variables were stable over the one-year period. While educational attainment tends to be stable across time, other characteristics like self-reported health or household income may not be. In a similar vein, there may be errors related to recalling outcomes in the past 12 months or bias arising from telescoping. These would manifest in misclassification of either predictors or outcomes. Previous studies using self-reported survey data addressed a potential underestimation of the number of experiences of unmet needs [8].

Using a secondary data source, this study was limited by the data that was collected. It can be acknowledged that a wide range of factors that were not included could also impact unmet need, such as provider characteristics. This study also did not have information on the types of health care services that were not received: it is unclear whether unmet needs arose from primary care services, hospital services, or both. It may be necessary to examine unmet health care need with a specific service rather than general experience of unmet health care need as a micro-level investigation focusing on specific health care services is suggested [9, 11, 12]. Instead of adopting a micro-level approach, this study adapted a classification of unmet needs from previous studies [11, 12], and attempted to understand the effect of various SES factors on different types of unmet health care needs. It should also be mentioned that there are two different ways to measure unmet health care needs. Based on clinical assessment, individuals who do not receive appropriate care can be determined as having unmet needs, as well as individuals who self-report their experiences of failing to receive appropriate care when needed. This study used the latter of the two approaches and may overlook the clinical factors associated with unmet health care needs at the individual level. However, previous studies suggest that self-reported experiences of unmet needs can be an appropriate measure when analyzing national population-based surveys [8]. Despite these limitations, the results provide notable information on the experiences of unmet health care need at the population level using a large, representative population-based survey, building on previous research.

## Conclusions

This study builds upon previous research by more deeply exploring how socioeconomic factors are associated with unmet needs and the sub-categories of unmet needs-availability, acceptability and accessibility. This study found that women of lower income had a higher level of experiencing overall unmet health care needs. The determinants of unmet need varied depending on the underlying reasons for such an unmet need. Lower income and lower educational level in both women and men increased likelihood of having accessibility-related unmet needs, while younger age groups and women living alone were associated with higher odds of having availability-related unmet needs. Lastly, individuals living in the southeastern regions (i.e., Busan, Daegu, Ulsan, and Gyeonsang province) were more likely to experience overall unmet health care needs. As unmet health care needs are a critical indicator of the health care system in a country, it is important to eliminate any obstacles that prevent access to, or limit the utilization of, health care services. Under the current universal health care system in Korea, women, in particular those of lower income and lower educational levels, have limited accessibility to necessary health care services. A gender-specific health care plan is suggested to reduce the high rate of unmet needs in this group. To reduce accessibility-related unmet needs, increasing services available to the younger age groups needs to be considered.

## Abbreviations

HBM: Health Behaviour Model
KNHANES: Korean National Health and Nutrition Examination Survey
NHI: National Health Insurance
OECD: Organisation for Economic Cooperative Development
OOP: Out of Pocket
OR: Odds Ratio
SES: Socioeconomic Status
